# Supplementary material for: NTS Prlh overcomes orexigenic stimuli and ameliorates dietary and genetic forms of obesity
Source: Nat Commun. 2021 Aug 30;12:5175. doi: 10.1038/s41467-021-25525-3 (PMC8405610; doi:10.1038/s41467-021-25525-3)
Supplement: Supplementary file 1 — Supplementary Information [file 41467_2021_25525_MOESM1_ESM.pdf]

Supplemental Figures

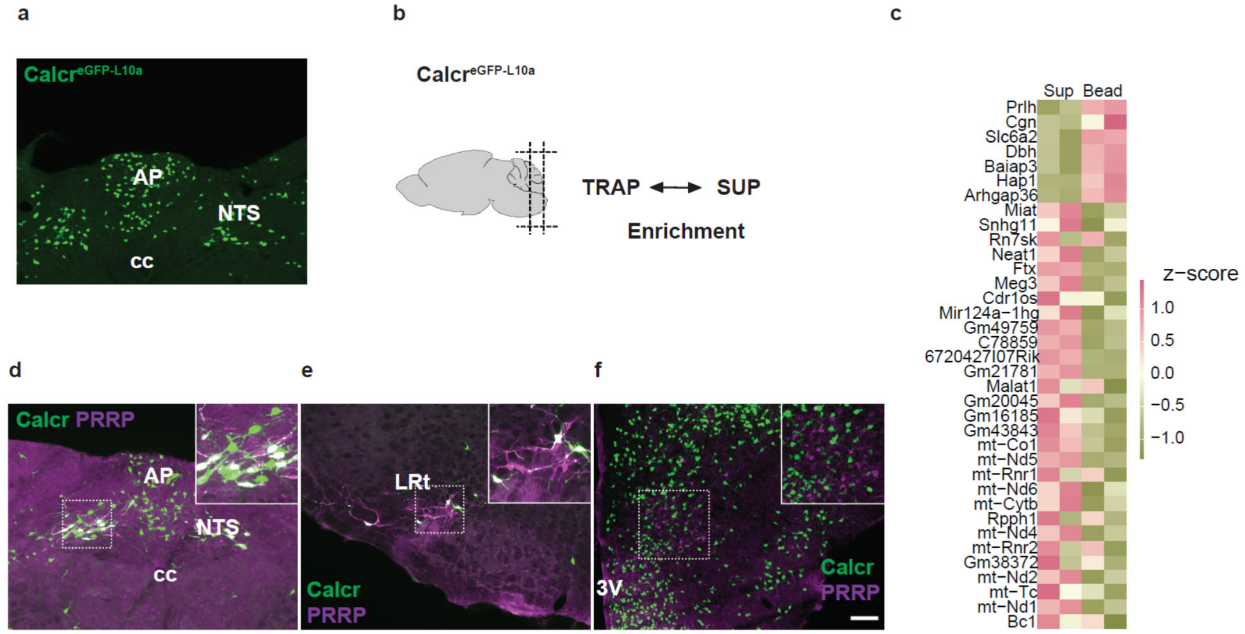

Supplemental figure 1: Expression of *Prlh* in Calcr neurons. **(A) I**mage showing GFP-IR (green) in the NTS and AP of a Calcr$^{eGFP\text{-}L10a}$ mouse; representative of at least three independent experiments with similar results. **(B)** Schematic diagram showing hindbrain dissection for TRAP and the comparison of TRAP-derived and supernatant mRNAs to determine enrichment. **(C)** Heat map showing genes, including *Prlh*, that are enriched in hindbrain *Calcr* neurons. Experiment was performed twice with one sample per condition; results, which were similar between experiments, were combined. (**D-F**) Representative images showing PRRP-IR (magenta) and GFP-IR (green) in PRRP-containing brain regions from a Calcr$^{eGFP\text{-}L10a}$ mouse: NTS **(D)**, LRt **(E)** and DMH **(F)**. Representative of at least three independent experiments with similar results. All images acquired at the same magnification; scale bar equals 150 µm. cc: central canal; 3v: third ventricle.

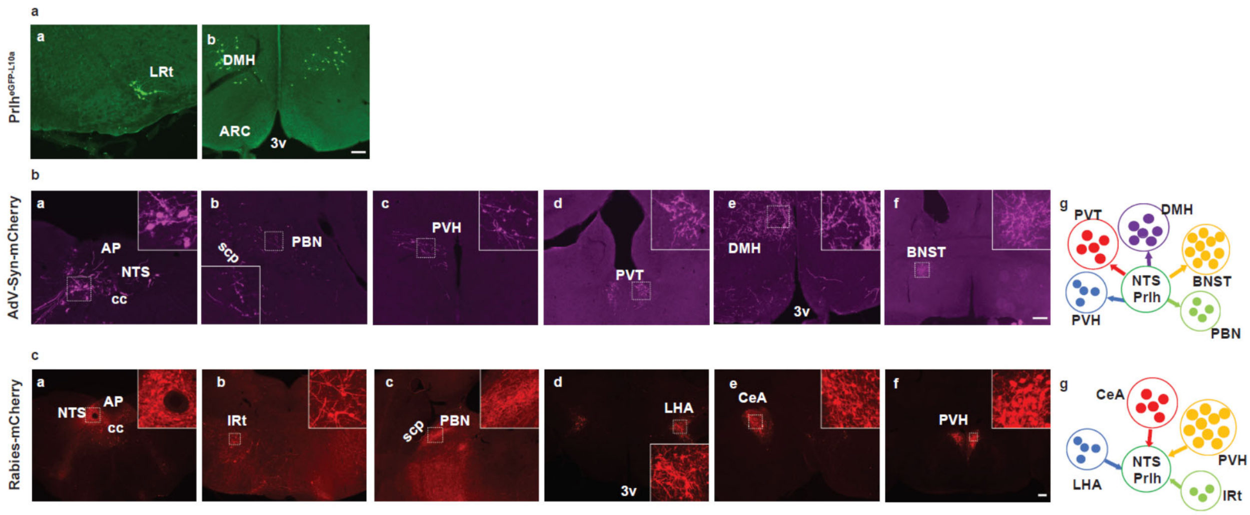

Supplemental figure 2: Distribution of *Prlh*^{cre}-mediated recombination and of afferent and efferent connections of Prlh^{NTS} cells. (**A**) Representative images of GFP-IR (green) in Prlh$^{eGFP\text{-}L10a}$ mice in the LRt (**Aa**) and DMH (**Ab**). (**B**) Representative images showing the distribution of dsRed-IR (purple) in the brain of *Prlh*^{cre} mice following the intra-NTS injection of AdV-Syn-mCherry (which mediates the cre-dependent expression of a synaptophysin-mCherry fusion protein), showing the NTS injection site (**Ba**) and projections fields in the PBN (**Bb**), PVH (**Bc**), PVT (**Bd**), DMH (**Be**) and BNST(**Bf**). Model of all projections is shown, with relative strength of innervation, in **Bg**. (**C**) AAV$^{Flex\text{-}TVA+G}$ was injected into the NTS of *Prlh*^{cre} mice; 1 month later the mice were injected in the NTS with pseudotyped mCherry-expressing rabies virus. Representative images show the injection site in the NTS (**Ca**), and traced cells in the LRt (**Cb**), PBN (fibers only) (**Cc**), LHA (**Cd**), CeA (**Ce**), and PVH (**Cf**). Model of all regions with neurons presynaptic to Prlh^{NTS} cells, with relative strength of innervation, is shown in **Cg**. LRt: Leteral reticular nucleus. cc= central canal, scp=superior cerebellar peduncle, LHA-lateral hypothalamus, CeA=The central nucleus of the amygdala, PVH-para ventricular hypothalamus. BNST= Bed Nucleus of the Stria Terminalis. 3v- third

cerebral ventricle. All other abbreviations defined in the main text. Images are representative of at least 3 animals. All images acquired at the same magnification; scale bar equals 150 μm. All data representative of at least two independent experiments consisting of at least two similar replicates.

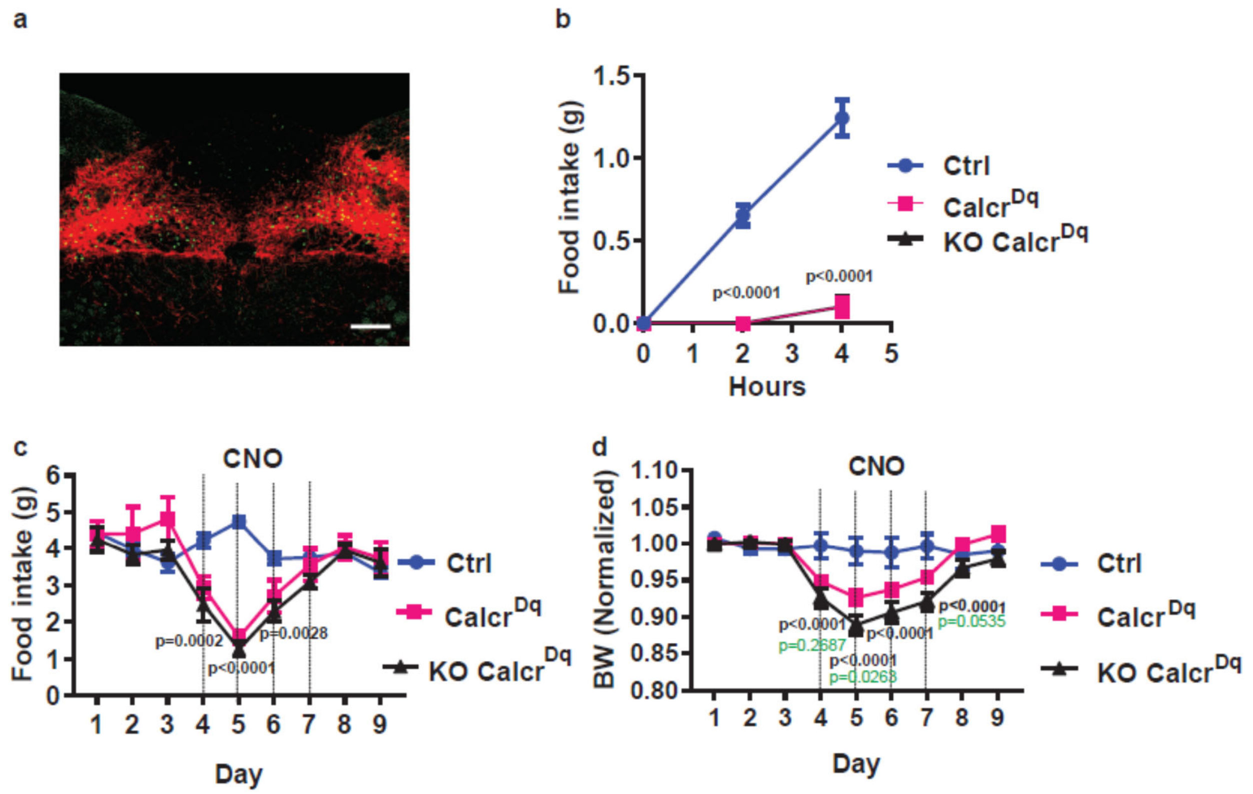

Supplemental figure 3: DREADD-mediated activation of Calcr^{NTS} neurons in Prlh^{Calcr}KO^{Dq} mice suppresses food intake despite lack of *Prlh* expression. (A) Representative image showing dsRed-IR (red) and FOS-IR (green) in CNO-treated (IP, 1 mg/kg, 2 hr) Prlh^{Calcr}KO^{Dq} mice. Scale bar equals 150 µm. (**B-D**) Food intake at the onset of the dark cycle during CNO (1 mg/kg, IP) treatment (**B,** n=15 Ctrl, n=7 Calcr^{Dq}, and n=8 KO Calcr^{Dq} animals) and food intake (**C,** n=7 Ctrl, n=6 Calcr^{Dq}, and n=8 KO Calcr^{Dq} animals) and body weight (**D**, normalized to initial weight, n=5 Ctrl, n=7 Calcr^{Dq}, and n=8 KO Calcr^{Dq} animals) for control (Ctrl, blue), Calcr$^{NTS\text{-}Dq}$ (Calcr^{Dq}, red) and Prlh^{Calcr}KO^{Dq} (KO Calcr^{Dq}, black) mice during treatment with vehicle (3 days), CNO (IP, 1 mg/kg BID; 4 days) and washout (2 days). Days of CNO treatment are marked on the graphs in **C, D**. Shown is mean +/- SEM. Two-way ANOVA, sidak's multiple comparisons test was used. Significant or near significant p values for comparisons

between Ctrl and KO Calcr^{Dq} or Calcr^{Dq} groups shown in black; those for comparisons between between KO Calcr^{Dq} and Calcr^{Dq} groups shown in green. All experiments were repeated in two independent cohorts of animals with similar results; cohorts were combined for publication.

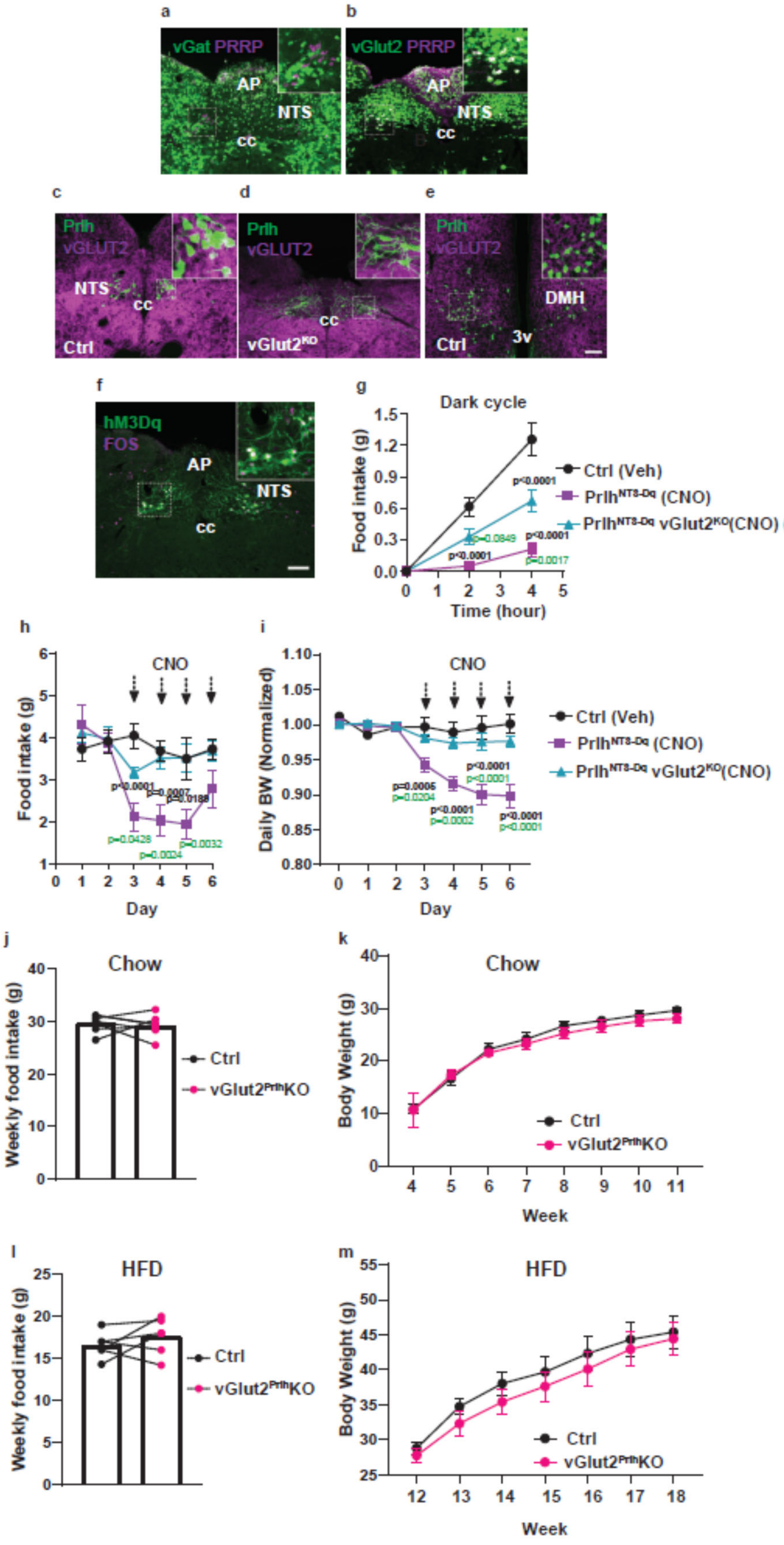
a
vGat PRRP
AP
NTS
cc
b
vGlut2 PRRP
AP
NTS
cc
c
Prlh
vGLUT2
NTS
cc
Ctrl
d
Prlh
vGLUT2
cc
vGlut2KO
e
Prlh
vGLUT2
DMH
Ctrl
3v
f
hM3Dq
FOS
AP
NTS
cc
g
Dark cycle
Food intake (g)
Time (hour)
Ctrl (Veh)
PrlhNTS-Dq (CNO)
PrlhNTS-Dq vGlut2KO(CNO)
h
CNO
Food intake (g)
Day
i
CNO
Daily BW (Normalized)
Day
Ctrl (Veh)
PrlhNTS-Dq (CNO)
PrlhNTS-Dq vGlut2KO(CNO)
j
Chow
Weekly food intake (g)
Ctrl
vGlut2PrlhKO
k
Chow
Body Weight (g)
Week
Ctrl
vGlut2PrlhKO
l
HFD
Weekly food intake (g)
Ctrl
vGlut2PrlhKO
m
HFD
Body Weight (g)
Week
Ctrl
vGlut2PrlhKO

Supplemental figure 4: Glutamatergic transmission contributes to the suppression of feeding during DREADD-mediated activation of Prlh^{NTS} neurons in Prlh^{Calcr}KO^{Dq} mice. (A, B) Representative images showing PRRP-IR (purple) and GFP-IR (green) in the NTS of vGat^{GFP} (**A**) and vGlut2^{GFP} (**B**) reporter mice. (**C-E**) Representative images showing GFP-IR (green, **C, E**) and PPRP-IR (green, **D**) and vGLUT2-IR (purple) in the NTS (**C, D**) and DMH (**E**) of Prlh$^{eGFP\text{-}L10a}$ (Ctrl; **C, E**) and *Prlh^{cre};vGlut2$^{flox/flox}$* (vGlut2^{KO}, **D**) mice. (**F**) Representative image showing dsRed-IR (green, hM3Dq) and FOS-IR (purple) in a CNO (IP, 1 mg/kg)-treated vGlut2^{KO} mouse that was injected with AAV$^{Flex\text{-}hm3dq}$ in the NTS (Prlh$^{NTS\text{-}Dq}$;vGlut2^{KO}). (**G-I**) Food intake at the onset of the dark cycle during CNO (1 mg/kg, IP) (**G**, n=12 Ctrl animals, n=10 Prlh$^{NTS\text{-}Dq}$, and n=7 Prlh$^{NTS\text{-}Dq}$;vGlut2^{KO} animals) and food intake (**H,** n=6 animals per group for Ctrl and Plrh$^{NTS\text{-}Dq}$;vGlut2^{KO}, n=7 for Prlh$^{NTS\text{-}Dq}$ animals) and body weight (**I**, normalized to initial weight, n=6 animals per group for Ctrl and Plrh$^{NTS\text{-}Dq}$;vGlut2^{KO}, n=7 for Prlh$^{NTS\text{-}Dq}$ animals) for control (Ctrl, black), Prlh$^{NTS\text{-}Dq}$ (purple) or Prlh$^{NTS\text{-}Dq}$;vGlut2^{KO} (blue) mice during treatment with vehicle (3 days), and then CNO (IP, 1 mg/kg BID; 4 days). Days of CNO treatment are marked on the graphs in **H, I**. (**J-M**) The 11th week food intake (**J,** n=7 animals/group) and weekly body weight (**K,** n=8 animals/group) of the control (black) and vGlut2^{KO} (red) mice fed with chow, and the 13rd week food (**L,** n=6 animals/group) and weekly body weight (**M,** n=7 animals/group) of the control and vGlut2^{KO} mice fed with HFD, all were male. Shown is mean +/- SEM; n=7-12 per group. Two-way ANOVA, sidak's multiple comparisons test was used. Significant or near significant p values for comparisons between Ctrl and Prlh$^{NTS\text{-}Dq}$ or Prlh$^{NTS\text{-}Dq}$;vGlut2^{KO} shown in black; those for comparisons between Prlh$^{NTS\text{-}Dq}$ and Prlh$^{NTS\text{-}Dq}$;vGlut2^{KO}

groups shown in green. All images acquired at the same magnification; scale bar equals 150 μm. All experiments were repeated in two independent cohorts of animals with similar results; cohorts were combined for publication.

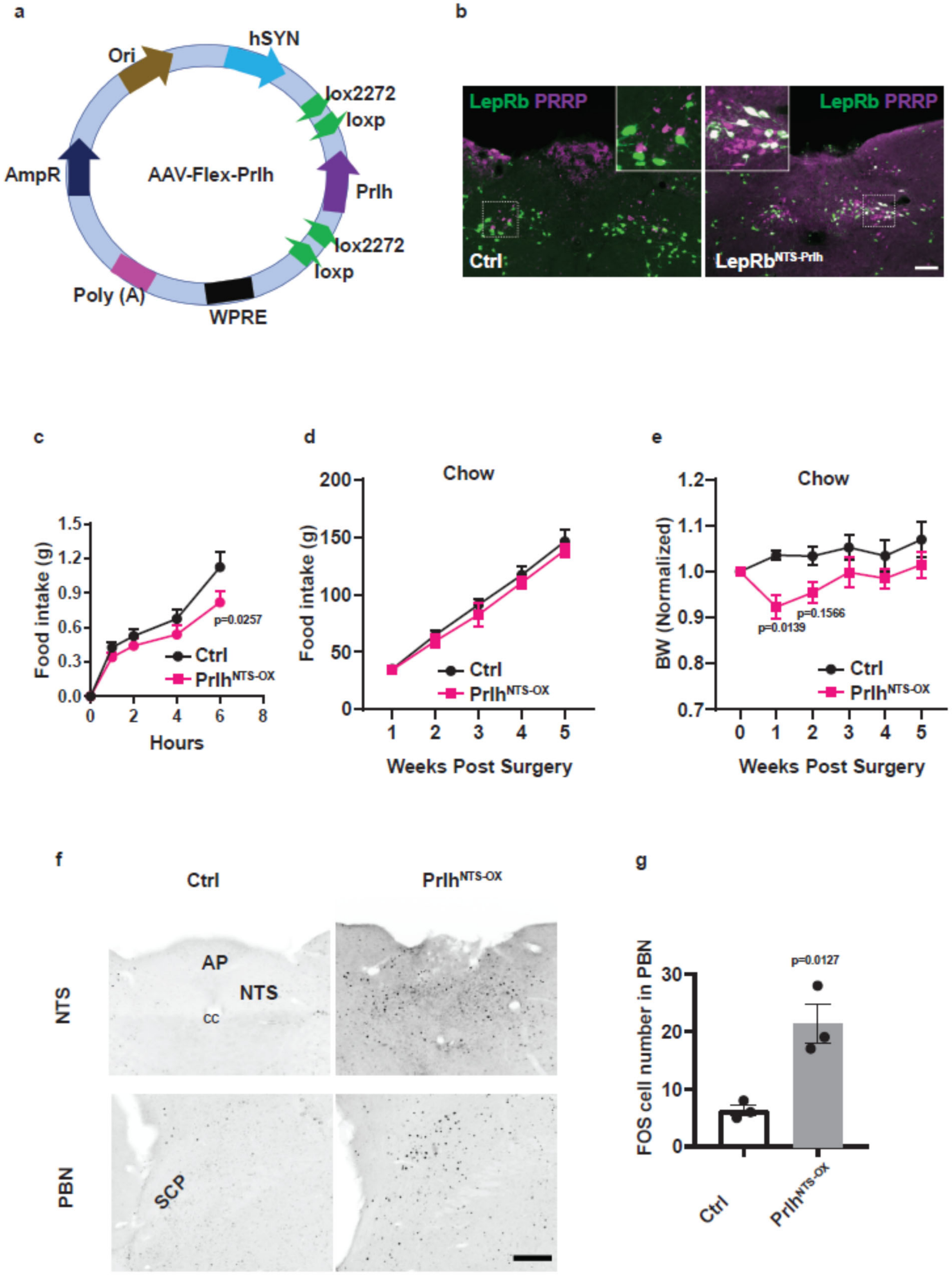
a
b
c
d
e
f
g
Ori
hSYN
lox2272
loxp
AAV-Flex-Prlh
AmpR
Prlh
lox2272
loxp
Poly (A)
WPRE
LepRb PRRP
Ctrl
LepRb PRRP
LepRbNTS-Prlh
Food intake (g)
1.5
1.2
0.9
0.6
0.3
0.0
0
2
4
6
8
Hours
p=0.0257
Ctrl
PrlhNTS-OX
Chow
Food intake (g)
200
150
100
50
0
1
2
3
4
5
Weeks Post Surgery
Ctrl
PrlhNTS-OX
Chow
BW (Normalized)
1.2
1.1
1.0
0.9
0.8
0.7
0
1
2
3
4
5
Weeks Post Surgery
p=0.0139
p=0.1566
Ctrl
PrlhNTS-OX
Ctrl
PrlhNTS-OX
NTS
AP
NTS
cc
PBN
SCP
FOS cell number in PBN
30
20
10
0
p=0.0127
Ctrl
PrlhNTS-OX

Supplemental figure 5: Effects of *Prlh* overexpression in Prlh^{NTS} neurons in lean mice. (**A**) Schematic showing AAV$^{Flex\text{-}Prlh}$ to mediate the cre-dependent overexpression of *Prlh*. **(B)** Representative images showing PRRP-IR (magenta) and GFP-IR (LepRb, green) in the NTS of LepRb$^{eGFP\text{-}L10a}$ control (Ctrl, left panel) or mice injected with AAV$^{Flex\text{-}Prlh}$ into the NTS (LepRb$^{NTS\text{-}Prlh}$, right panel). **(C)** Food intake during daytime refeeding following an overnight fast for chow-fed control (Ctrl, black) and Prlh$^{NTS\text{-}OX}$ (red) mice. (**D, E**) Cumulative weekly food intake (**D,** n=4 control and n=5 Prlh$^{NTS\text{-}OX}$ animals/group) and weekly body weight (**E**, normalized to initial weight, n=4 control and n=6 Prlh$^{NTS\text{-}OX}$ animals/group) over the five weeks following AAV injection in chow-fed control (Ctrl) and Prlh$^{NTS\text{-}OX}$ mice. Shown is mean +/- SEM; n=4-7 per group. Two-way ANOVA, sidak's multiple comparisons test was used. Significant or near significant p values are shown for comparisons between Ctrl and Prlh$^{NTS\text{-}OX}$ groups. (**F**) Representative images of FOS-IR (black) in the NTS (top panels) and PBN (bottom panels) of control (Ctrl) and Prlh$^{NTS\text{-}OX}$ mice, n=7 animals/group. All images were acquired at the same magnification; scale bar equals 150 μm. cc= central canal, scp=superior cerebellar peduncle. (**G**) PBN FOS-IR cell counts for control (Ctrl) and Prlh$^{NTS\text{-}OX}$ mice. Shown is mean +/- SEM; n=3 per group Significant or near significant p values by unpaired t-test are shown for comparisons between Ctrl and Prlh$^{NTS\text{-}OX}$ groups. All experiments were repeated in two independent cohorts of animals with similar results; cohorts were combined for publication.

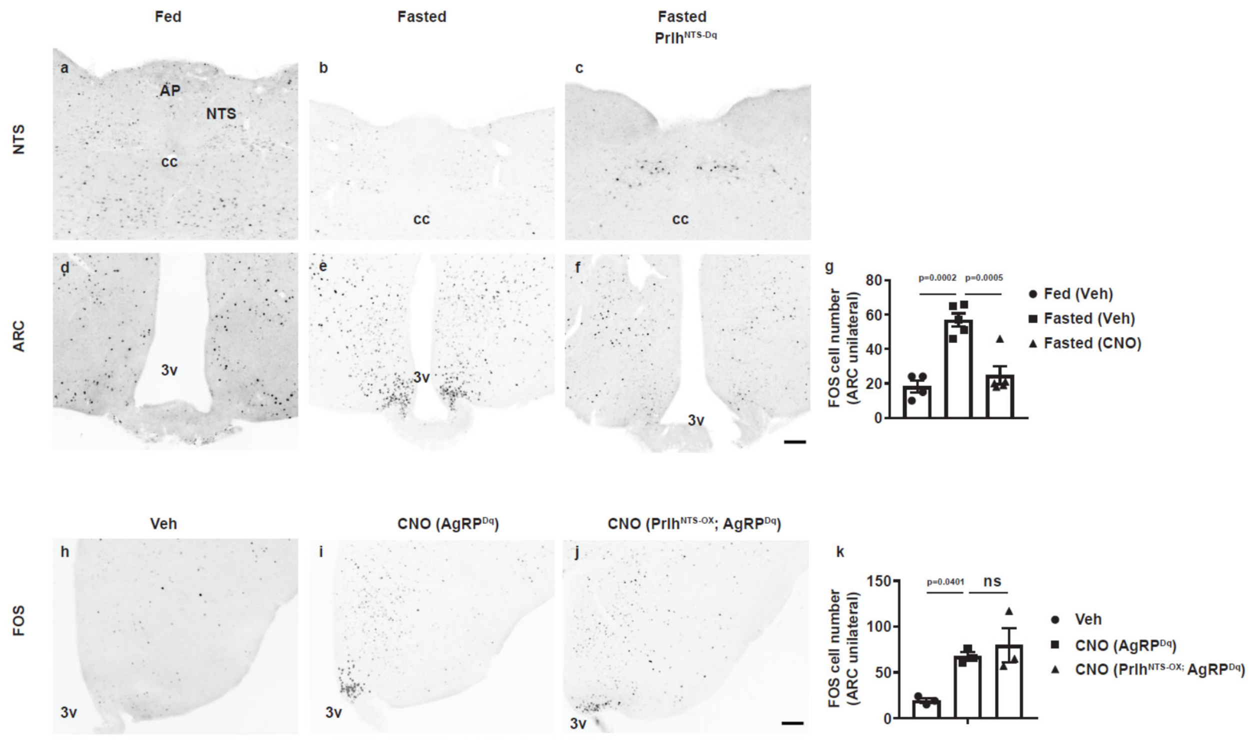

Supplemental figure 6: Suppression of mbARC FOS by Prlh^{NTS} neurons and activation of AgRP neurons in Prlh^{NTS-OX};Agrp^{Dq} mice. (A-G) Representative images showing FOS-IR (black) in the NTS (**A-C**) and ARC (**D-F**) of *ad libitum*-fed control animals (Fed; **A, D**), overnight fasted control animals (Fasted; **B, E**), overnight fasted CNO-treated (IP, 1 mg/kg, 2 hours) Prlh^{NTS-Dq} mice (Fasted Prlh^{NTS-Dq}; **C, F**) and FOS positive cell counts in ARC of mice in the condition of being fed (n=4 animals), fasted (n=5 animals) and fasted with CNO injection (IP, 1 mg/kg) (n=5 animals) (**G**). Shown is mean +/- SEM; One-way ANOVA, Dunnett's multiple comparisons test was used. Significant or near significant p values are shown for comparisons to Fasted (Veh) group. (**H-J**) Representative images showing FOS-IR (black) in the ARC of vehicle (Veh) or CNO-treated (IP, 1 mg/kg) Agrp^{Dq} or Prlh^{NTS-OX};AgRP^{Dq} mice. (**K**) Quantification of mbARC fos in mice treated as in H-J; n=3. cc= central canal. 3v: third ventricle. Shown is mean +/- SEM; One-way ANOVA, Dunnett's multiple comparisons

test was used. Significant or near significant p values are shown for comparisons to CNO ($AgRP^{Dq}$) group. ns: not significant ($p>0.05$) vs. $AgRP^{Dq}$. All images taken at same magnification, scale bars= 150 μm. Results shown in the images are representative of at least two independent experiments; quantification was done on one experiment.
