# Supplementary material for: NTS Prlh overcomes orexigenic stimuli and ameliorates dietary and genetic forms of obesity
Source: Nat Commun. 2021 Aug 30;12:5175. doi: 10.1038/s41467-021-25525-3 (PMC8405610; doi:10.1038/s41467-021-25525-3)
Supplement: Supplementary file 3 — Reporting Summary [file 41467_2021_25525_MOESM3_ESM.pdf]

nature research

Corresponding author(s): Wenwen Cheng and Martin G Myers, Jr.

Last updated by author(s): Aug 3, 2021

Reporting Summary

Nature Research wishes to improve the reproducibility of the work that we publish. This form provides structure for consistency and transparency in reporting. For further information on Nature Research policies, see our Editorial Policies and the Editorial Policy Checklist.

Statistics

For all statistical analyses, confirm that the following items are present in the figure legend, table legend, main text, or Methods section.

n/a	Confirmed	
[ ]	[x]	The exact sample size (*n*) for each experimental group/condition, given as a discrete number and unit of measurement
[ ]	[x]	A statement on whether measurements were taken from distinct samples or whether the same sample was measured repeatedly
[ ]	[x]	The statistical test(s) used AND whether they are one- or two-sided<br>*Only common tests should be described solely by name; describe more complex techniques in the Methods section.*
[x]	[ ]	A description of all covariates tested
[x]	[ ]	A description of any assumptions or corrections, such as tests of normality and adjustment for multiple comparisons
[ ]	[x]	A full description of the statistical parameters including central tendency (e.g. means) or other basic estimates (e.g. regression coefficient) AND variation (e.g. standard deviation) or associated estimates of uncertainty (e.g. confidence intervals)
[ ]	[x]	For null hypothesis testing, the test statistic (e.g. *F*, *t*, *r*) with confidence intervals, effect sizes, degrees of freedom and *P* value noted<br>*Give P values as exact values whenever suitable.*
[x]	[ ]	For Bayesian analysis, information on the choice of priors and Markov chain Monte Carlo settings
[x]	[ ]	For hierarchical and complex designs, identification of the appropriate level for tests and full reporting of outcomes
[x]	[ ]	Estimates of effect sizes (e.g. Cohen's *d*, Pearson's *r*), indicating how they were calculated

Our web collection on statistics for biologists contains articles on many of the points above.

Software and code

Policy information about availability of computer code

Data collection	*Provide a description of all commercial, open source and custom code used to collect the data in this study, specifying the version used OR state that no software was used.*
Data analysis	fastp 0.21.0; STAR 2.7.7a; DEseq2 1.20.1; Photoshop 2020; Image J; PRISM version 8

For manuscripts utilizing custom algorithms or software that are central to the research but not yet described in published literature, software must be made available to editors and reviewers. We strongly encourage code deposition in a community repository (e.g. GitHub). See the Nature Research guidelines for submitting code & software for further information.

Data

Policy information about availability of data

All manuscripts must include a data availability statement. This statement should provide the following information, where applicable:

- Accession codes, unique identifiers, or web links for publicly available datasets
- A list of figures that have associated raw data
- A description of any restrictions on data availability

All raw data will be made available upon request; data for all graphs is supplied in the supplementary information/source data file. TRAP-seq data are available at GEO: GSE176202; https://www.ncbi.nlm.nih.gov/geo/query/acc.cgi?acc=GSE176202.

Field-specific reporting

Please select the one below that is the best fit for your research. If you are not sure, read the appropriate sections before making your selection.

☒ Life sciences ☐ Behavioural & social sciences ☐ Ecological, evolutionary & environmental sciences

For a reference copy of the document with all sections, see nature.com/documents/nr-reporting-summary-flat.pdf

Life sciences study design

All studies must disclose on these points even when the disclosure is negative.

Sample size	Sample sizes were generally 5-10 for measurements involving body weight, food intake, adiposity, as our previous experience has demonstrated this sample size to provide a robust assessment of difference in these measures (See references 4, 5, 15, 16, 20, 22, 29, 37).
Data exclusions	Animals whose injections missed the intended target, as revealed by IHC, were excluded.
Replication	All experiments were repeated in two independent cohorts of animals with similar results; cohorts were generally combined for publication.
Randomization	All experiments involved animals; animals were assigned to groups and treated in the order of ear tag numbers. Ear tag numbers were assigned randomly within a litter
Blinding	As a single investigator bred, genotyped, performed surgeries and recorded data from each experimental subject, blinding was not possible.

Reporting for specific materials, systems and methods

We require information from authors about some types of materials, experimental systems and methods used in many studies. Here, indicate whether each material, system or method listed is relevant to your study. If you are not sure if a list item applies to your research, read the appropriate section before selecting a response.

Materials & experimental systems

n/a	Involved in the study
☐	☒ Antibodies
☒	☐ Eukaryotic cell lines
☒	☐ Palaeontology and archaeology
☐	☒ Animals and other organisms
☒	☐ Human research participants
☒	☐ Clinical data
☒	☐ Dual use research of concern

Methods

n/a	Involved in the study
☒	☐ ChIP-seq
☒	☐ Flow cytometry
☒	☐ MRI-based neuroimaging

Antibodies

Antibodies used	FOS, #2250, Cell Signaling Technology, 1:1000; GFP, GFP1020, Aves Laboratories, 1:1000; dsRed, 632496, Takara, 1:1000; prolactin-releasing peptide (PRRP), H-008-52, Phoenix Pharmaceuticals, 1:500
Validation	Cell. 2017 Aug 24;170(5):1013-1027.e14. doi: 10.1016/j.cell.2017.07.020. Epub 2017 Aug 17. ;<br>https://www.aveslabs.com/products/anti-green-fluorescent-protein-antibody-gfp;<br>https://www.takarabio.com/products/antibodies-and-elisa/fluorescent-protein-antibodies/red-fluorescent-protein-antibodies;<br>Cell Metab. 2014 Oct 7;20(4):639-49.doi: 10.1016/j.cmet.2014.07.022. Epub 2014 Aug 28.

Animals and other organisms

Policy information about studies involving animals; ARRIVE guidelines recommended for reporting animal research

Laboratory animals	mouse lines are as described in methods; all animals were on the segregating C57BL6;SJLF2 background
Wild animals	no wild animals were used in this study
Field-collected samples	no filed-collected animals were used in this study
Ethics oversight	Approval by University of Michigan IACUC, and care was according to NIH and AALAC-approved guidelines.

Note that full information on the approval of the study protocol must also be provided in the manuscript.
